# Serum Albumin Is Independently Associated with Persistent Organ Failure in Acute Pancreatitis

**DOI:** 10.1155/2017/5297143

**Published:** 2017-09-24

**Authors:** Wandong Hong, Suhan Lin, Maddalena Zippi, Wujun Geng, Simon Stock, Zarrin Basharat, Bicheng Cheng, Jingye Pan, Mengtao Zhou

**Affiliations:** ^1^Department of Gastroenterology and Hepatology, The First Affiliated Hospital of Wenzhou Medical University, Wenzhou, Zhejiang, China; ^2^Unit of Gastroenterology and Digestive Endoscopy, Sandro Pertini Hospital, Rome, Italy; ^3^Department of Anesthesiology, The First Affiliated Hospital of Wenzhou Medical University, Wenzhou, Zhejiang, China; ^4^Department of Surgery, World Mate Emergency Hospital, Battambang, Cambodia; ^5^Microbiology & Biotechnology Research Lab, Department of Environmental Sciences, Fatima Jinnah Women University, Rawalpindi 46000, Pakistan; ^6^Zhejiang Provincial Top Key Discipline in Surgery, Wenzhou Key Laboratory of Surgery, Department of Surgery, The First Affiliated Hospital, Wenzhou Medical University, Wenzhou, Zhejiang, China; ^7^Intensive Care Unit, The First Affiliated Hospital of Wenzhou Medical University, Wenzhou, Zhejiang, China; ^8^Department of Surgery, The First Affiliated Hospital of Wenzhou Medical University, Wenzhou, Zhejiang, China

## Abstract

**Background and Aims:**

To investigate the association between serum albumin levels within 24 hrs of patient admission and the development of persistent organ failure in acute pancreatitis.

**Methods:**

A total of 700 patients with acute pancreatitis were enrolled. Multivariate logistic regression and subgroup analysis determined whether decreased albumin was independently associated with persistent organ failure and mortality. The diagnostic performance of serum albumin was evaluated by the area under Receiver Operating Characteristic (ROC) curves.

**Results:**

As levels of serum albumin decrease, the risk of persistent organ failure significantly increases (*P*_trend_ < 0.001). The incidence of organ failure was 3.5%, 10.6%, and 41.6% in patients with normal albumin and mild and severe hypoalbuminaemia, respectively. Decreased albumin levels were also proportionally associated with prolonged hospital stay (*P*_trend_ < 0.001) and the risk of death (*P*_trend_ < 0.001). Multivariate analysis suggested that biliary etiology, chronic concomitant diseases, hematocrit, blood urea nitrogen, and the serum albumin level were independently associated with persistent organ failure. Blood urea nitrogen and the serum albumin level were also independently associated with mortality. The area under ROC curves of albumin for predicting organ failure and mortality were 0.78 and 0.87, respectively.

**Conclusion:**

A low serum albumin is independently associated with an increased risk of developing of persistent organ failure and death in acute pancreatitis. It may also be useful for the prediction of the severity of acute pancreatitis.

## 1. Introduction

Though most patients with acute pancreatitis (AP) have a benign clinical course, approximately 10%–20% of patients develop persistent organ failure (defined as organ failure lasting for ≥48 hours) associated with significant mortality of at least 30% [1, 2]. Recent international consensus identified that persistent organ failure is the key determinant of severity regardless of the presence or absence of local pancreatic complications [3]. Early identification of patients likely to develop persistent organ failure would help physicians to select those patients who would benefit the most from close surveillance or aggressive intervention [4]. Existing scoring system such as the Bedside index of severity in acute pancreatitis (BISAP) [5] and the Harmless acute pancreatitis score (HAPS) [6] have moderate diagnostic accuracy in the prediction of persistent organ failure [2]. More recently, attention has also focused on assessing the association between persistent organ failure in acute pancreatitis and individual laboratory parameters such as admission hematocrit ≥ 44%, rise in blood urea nitrogen (BUN) at 24 hours [7], serum triglycerides [8], and high-density lipoprotein [9].

Albumin is exclusively synthesized in the liver. Low serum albumin (<35 g/L) is often observed in hospitalized elderly patients [10] or patients with decompensated liver cirrhosis, malnutrition, nephrotic syndrome, diabetes, heart failure, cancer, and sepsis [11, 12]. Hypoalbuminemia has been proposed as a useful predictor of morbidity and mortality in many different clinical settings such as community-acquired pneumonia [12] and hemodialysis and coronary heart disease [13]. As a negative acute phase protein, initial serum albumin was also independently associated with disease progression to severe sepsis [14] and 30-day mortality in emergency medical patients, irrespective of the cause [15, 16].

A few studies have evaluated hypoalbuminemia as predictor of severe acute pancreatitis [17–19]. However, these studies are limited due to small sample size with low statistical power and inconsistent definitions of severity ranging from severe pancreatitis based on the Atlanta criteria to mortality. Although a recent study by Li et al. [20] had used persistent organ failure of acute pancreatitis as the primary outcome, the results of that study [20], as well as the above-mentioned studies [17–19], are biased due to lack of adjustment for confounding factors such as etiology and chronic concomitant diseases. Furthermore, to the best of our knowledge, no study has performed a complete overview of the association between serum albumin levels at admission and outcomes in acute pancreatitis up until now. Therefore, the aim of this study was to investigate whether serum albumin levels within 24 hrs of patient admission correlate with clinical outcomes in acute pancreatitis.

## 2. Materials and Methods

Patients with AP who were admitted (index admissions) to our hospital within 72 hours of the onset of symptoms between January 2012 and January 2015 were enrolled for the study. Acute pancreatitis was defined as described previously [4]. Organ failure [1] was defined as having a Marshall score ≥ 2 for at least one of the three organs (respiratory, cardiovascular, and renal failure) involved. Duration of organ failure was defined as persistent if it lasted for >48 hours [1, 3]. Exclusion criteria included [21] patients that had developed organ failure before data collection, recurrent or not first-time pancreatitis, previous pancreatic surgery, ERCP or trauma-induced pancreatitis, chronic pancreatitis, pancreatic cancer, pleural effusions preceding the development of AP, and pleural effusions resulting from concomitant diseases (e.g., pneumonia, chronic heart failure), patients with albumin infusion before data collection in our hospital, hypoalbuminemia due to malnutrition, chronic renal disease, albuminuria, hepatitis, bleeding/coagulation disorders, chronic alcoholism, and liver cirrhosis, and patients for whom completed data was unavailable. Chronic concomitant diseases [12] were classified as neurologic (stroke), cardiovascular (coronary heart disease and arrhythmia), pulmonary (emphysema and chronic bronchitis), diabetes mellitus, hypertension, hepatitis virus carrier, and fatty liver. This study protocol was approved by the Ethics Committee of the First Affiliated Hospital of Wenzhou Medical College.

Age, gender, body mass index (BMI), time from pain onset to admission, and biochemical parameters were recorded within 12 hours of admission before the development of persistent organ failure [4, 21]. Serum albumin levels were measured within 24 hours of admission [12]. If patients had multiple albumin measurements within 24 hrs, only the first-time measurement was picked. Hypoalbuminemia was defined by a serum albumin < 35 g/l [11]. Similar to previous studies [8, 22], patients with hypoalbuminemia were further divided into mild (<35 g/l but ≥30 g/l) and severe (<30 g/l) groups according to the serum albumin level.

### 2.1. Statistical Analysis

Sample size calculation was based on identifying independent dichotomous predictors in a multivariable logistic regression analysis for persistent organ failure [23]. With an *α* risk of 0.05 (*α* is the probability of rejecting a true null hypothesis) and *β* risk of 0.2 (*β* is the probability of accepting a false null hypothesis), and a bilateral test, the sample size was calculated for the following hypotheses: assuming a prevalence of persistent organ failure at 10% and a low correlation between the predictor and other covariates (*R*^2^ = 0.10) (*R*-squared, namely, the coefficient of determination, which is equal to the squared correlation coefficient and provides an estimate of the percent of variation in one variable that is explained by the other variables [24]). A sample of 668 patients would provide 80% power of detecting an adjusted odds ratio of 2.0 for a predictor with an overall prevalence of 35%.

A Shapiro-Wilk test was used to evaluate whether the continuous data was a normal distribution. According to the results of Shapiro-Wilk test, continuous values were expressed by mean ± standard deviation (SD) or median and interquartile range (IQR) and compared using one-way analysis of variance or the Kruskal-Wallis nonparametric test. Categorical values were described by count and proportions and compared by the *χ*^2^ test or Fisher's exact test.

Linear trend of categorical and continuous variables was tested by a Royston extension of the Cochran-Armitage test [25] and a nonparametric Wilcoxon rank sum test [26], respectively.

Univariate logistic regression analysis was used to identify potential independent predictors of persistent organ failure and death. Except for serum albumin level and the time from pain onset to admission [8], all other variables, namely, age (≥60 years) [8], male sex [27], BMI (≥30) [28], biliary etiology (yes versus no), chronic concomitant diseases (yes versus no) [12], hematocrit (≥44%) [7], total bilirubin (≥2 mg/dL), alanine aminotransferase (ALT > 50 units/L) [29], glucose (≥150 mg/dL) [28], and blood urea nitrogen (BUN) (>25 mg/dL) [5], were used as dichotomous/categorical variable when performing logistic regression analysis. Variables reaching statistical difference among patients with different albumin levels or between patients with and without organ failure in univariate logistic regression analysis were included in the multivariate logistic regression analysis. Multivariate logistic regression was used to evaluate the relationship between patients with different albumin levels and persistent organ failure and death adjusted for potential risk factors and confounding factors. Odds ratios (OR) were calculated, with 95% CI [30]. Multicollinearity was considered to be significant if the largest variance inflation factor exceeded 10 [4].

The area under the Receiver Operating Characteristic Curve (AUC) was used to evaluate the performance of predictions. The serum albumin level was used as a continuous rather than categorical variable when performing Receiver Operating Characteristic (ROC) curve analysis. A variable with an AUC above 0.7 was considered useful, while an AUC between 0.8 and 0.9 indicated excellent diagnostic accuracy [31]. As described by Maksimow et al. [32], the best cut-off point was selected based on that where the number of false positives is as low as possible (specificity > 90%) by selecting a threshold value at a point where the longest increase in the sensitivity of the slope declines since ICU beds are limited. The sensitivity, specificity, negative predictive value, positive predictive value, positive likelihood ratio, negative likelihood ratio, and diagnostic accuracy were calculated for corresponding cut-off values.

Differences were considered to be statistically significant if the two-tailed *P* value was less than 0.05.

## 3. Results

### 3.1. Characteristics of Study Subjects

A total of 700 patients were included in the study. 435 (62.1%) were male, with a median age of 48 (IQR 37–63) years. The mean time interval between onset and admission was 1.8 ± 0.8 days. The median value of albumin within 24 hrs of admission was 36.2 g/L (IQR: 32.9–39.7). The overall prevalence of hypoalbuminemia (<35 g/L) was 39.4% (276/700). Of these, 198 had mild hypoalbuminemia, while 78 had severe hypoalbuminemia.

In all the patients, the most common etiology of AP was biliary (42.7%). 24 patients had combined biliary and alcohol etiology and 9 patients had combined biliary and hypertriglyceridemia etiology. Nearly 57.4% (402/700) of patients had ≥1 comorbidities, mainly fatty liver (257/700, 36.7%), hypertension (160/700, 22.9%), and diabetes mellitus (105/700, 15.0%). 68 (9.7%) patients developed persistent OF, which was multiple (≥1) OF, in 31 (45.6%) patients. Respiratory failure (82.4%) was the most frequent OF. Eleven (1.6%) patients with OF died from AP, seven during the first week in hospital.

### 3.2. Clinical Features and Results of Patients with Different Serum Albumin Levels

The demographic, clinical, and laboratory findings at hospital admission of patients with different serum albumin levels are shown in Table 1. A greater increase in age (*P*_trend_ = 0.012) and longer time from pain onset to admission (*P*_trend_ < 0.001) were associated with decreased serum albumin level. No significant difference was observed among patients with different albumin levels with respect to gender, BMI, and alcohol etiology.

There was also a trend for increasing rates of overall incidence of chronic concomitant diseases with a lower serum albumin level though it did not reach statistical significance (*P* = 0.134). However, there was a significant statistical difference among patients with different albumin levels with respect to biliary etiology and concomitant neurologic disease though it occurred in a few patients. Regarding laboratory findings, decline in serum albumin level was associated with a decrease in hematocrit (*P* = 0.001), alanine aminotransferase (*P* = 0.001), glucose (*P* = 0.003), and BUN (*P* < 0.001).

**Figure 1:**
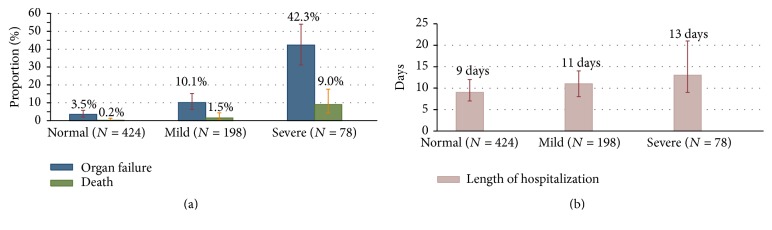
Trends of median hospital days, incidence of persistent organ failure, and mortality in acute pancreatitis patients with normal, mild, and severe low serum albumin.

As shown in Table 1 and Figure 1, persistent organ failure developed in 3.5% (15/424) patients with normal albumin, 10.1% (20/198) patients with mild hypoalbuminemia, and 42.3% (33/78) with severe hypoalbuminemia when patients were divided into three groups according to different albumin levels. This indicated that a greater decrease in serum albumin level was associated with increased organ failure rate (*P*_trend_ < 0.001). There was also a statistically significant trend for increasing rates of mortality (*P*_trend_ < 0.001) and prolonged hospital stay (*P*_trend_ < 0.001) with a lower serum albumin level.

### 3.3. Independent Predictors of Organ Failure and Mortality

**Figure 2:**
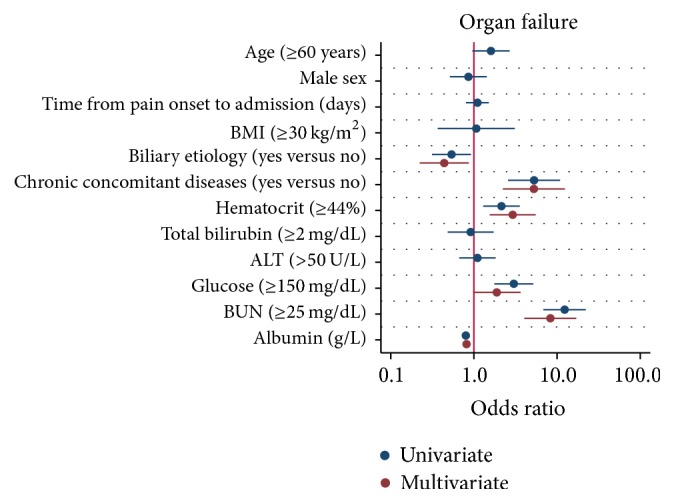
Univariate and multivariate logistic regression plot of odds ratios and 95% confidence intervals for evaluation of the relationship between serum albumin level and persistent organ failure in acute pancreatitis.

As shown in Figure 2, univariate analysis revealed that biliary etiology, chronic concomitant diseases, hematocrit, glucose, BUN, and the serum albumin level were significantly associated with the development of persistent organ failure. Multivariable logistic regression identified biliary etiology (OR: 0.44; 95% CI: 0.22–0.87; *P* = 0.018), chronic concomitant diseases (OR: 5.26; 95% CI: 2.07–11.31; *P* < 0.001), hematocrit ≥ 44% (OR: 2.92; 95% CI: 1.55–5.51; *P* = 0.001), BUN > 25 mg/dL (OR: 8.30; 95% CI: 4.05–17.01; *P* < 0.001), and the serum albumin level (OR: 0.81; 95% CI: 0.76–0.87; *P* < 0.001) were independently associated with persistent organ failure.

**Figure 3:**
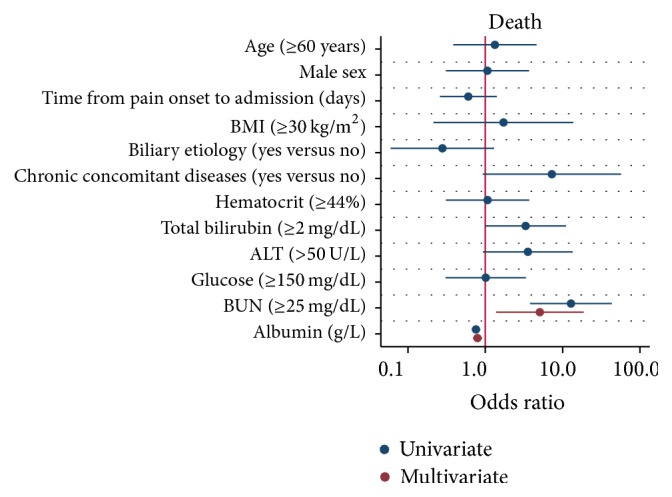
Univariate and multivariate logistic regression plot of odds ratios and 95% confidence intervals for evaluation of the relationship between serum albumin level and death in acute pancreatitis.

As shown in Figure 3, factors considered potentially relevant to mortality were analyzed by univariate and multivariate logistic regression. Multivariate logistic regression identified that BUN > 25 mg/dL (OR: 5.06; 95% CI: 1.37–18.67; *P* = 0.015) and the serum albumin level (OR: 0.79; 95% CI: 0.69–0.91; *P* = 0.001) were independently associated with mortality. Multicollinearity between covariates did not exist, and the variance inflation factor was less than 10 for all variables.

### 3.4. Subgroup Analysis

**Figure 4:**
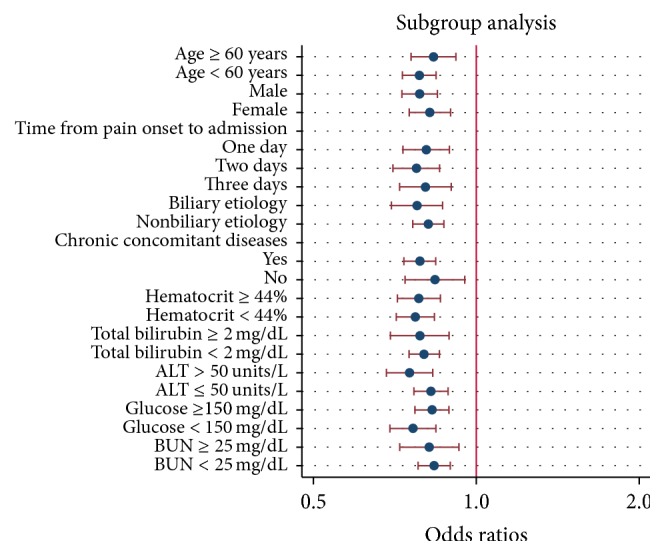
Logistic regression plot of odds ratios and 95% confidence intervals; subgroup analysis of relationship between serum albumin level and risk of persistent organ failure in acute pancreatitis.

**Figure 5:**
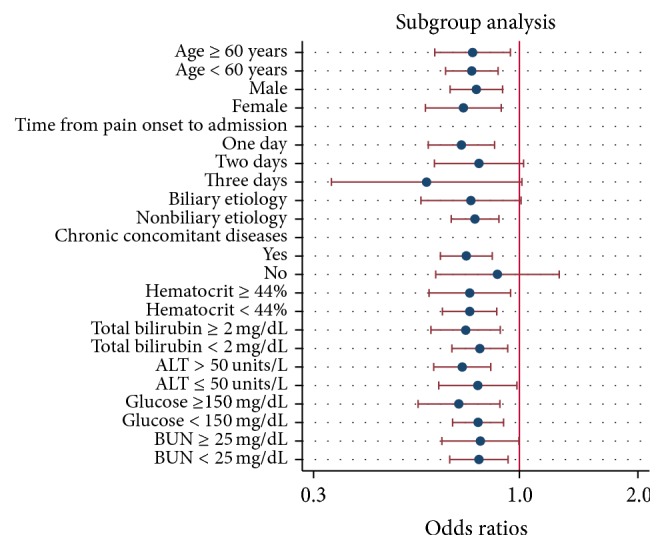
Logistic regression plot of odds ratios and 95% confidence intervals; subgroup analysis of relationship between serum albumin level and mortality in acute pancreatitis.

Subgroup analysis confirmed the strong association between the serum albumin level and persistent organ failure across subgroups of age, gender, the time from pain onset to admission, different etiology, chronic concomitant diseases, total bilirubin, ALT, hematocrit, glucose, and BUN (Figure 4). As to association between the serum albumin level and mortality, subgroup analysis confirmed that these associations were stronger in subgroups of patients with one day from pain onset to admission, nonbiliary etiology, and chronic concomitant diseases than those in subgroups with two or three days from pain onset to admission and biliary etiology and without chronic concomitant diseases (Figure 5).

### 3.5. Albumin as Early Predictor of Persistent Organ Failure and Mortality

**Figure 6:**
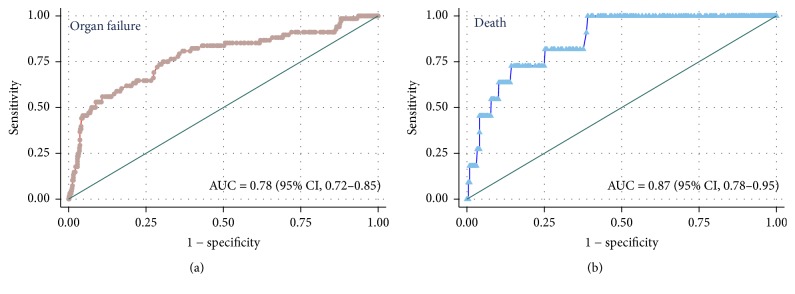
Receiver operating characteristic curves for serum albumin to predict the development of persistent organ failure and occurring of death in acute pancreatitis.

The ROC curve for albumin for the prediction of persistent organ failure and mortality is shown in Figure 6. The AUC of albumin for predicting organ failure and mortality were 0.78 (95% CI: 0.72–0.85) and 0.87 (95% CI: 0.78–0.95), respectively. As shown in Table 2, the best cut-off points to predict organ failure and mortality were albumin level ≤ 30.8 g/L and ≤29 g/L, respectively.

## 4. Discussion

The results of the present study demonstrated the following: (i) persistent organ failure and early hypoalbuminemia occurred in 9.7% and 39.3% of patients with acute pancreatitis, respectively; (ii) as a risk factor, low serum albumin levels within 24 hours of admission are independently associated with the development of persistent organ failure and mortality in acute pancreatitis. Furthermore, this association is proportional; meaning the lower the serum albumin level, the greater the likelihood for persistent organ failure and mortality. The associations between the serum albumin level and mortality were stronger in subgroups of patients with one day from pain onset to admission, nonbiliary etiology, and chronic concomitant diseases than those in subgroups with two or three days from pain onset to admission and biliary etiology and without chronic concomitant diseases; (iii) as a predictor, the serum albumin had moderate and excellent performance for prediction of persistent organ failure (AUC: 0.78) and mortality (AUC: 0.87) in acute pancreatitis, respectively.

The mechanisms of hypoalbuminemia in acute pancreatitis are diverse [11, 33, 34]: (i) a decrease in energy or amino acid supply due to fasting and increased tissue catabolism in acute pancreatitis, (ii) excessive release of inflammatory cytokines such as interleukin-1, interleukin-6, and tumor necrosis factor *α* in the early stage of acute pancreatitis resulting in a decreased albumin synthesis in the liver, and (iii) active inflammatory cytokines also injuring microvascular endothelial cells which result in increased capillary permeability leading to a redistribution of albumin from the intravascular to the interstitial space.

Albumin has many physiologic roles including maintaining osmotic pressure, protecting the microvasculature and mitigating increased vascular permeability, binding of endogenous and exogenous substances, antioxidant and scavenging free radicals, anticoagulant effect, maintenance of acid base status, anti-inflammatory effects, and antiapoptosis [12, 33]. Therefore, hypoalbuminemia may be causally linked to the development of persistent organ failure rather than serving simply as a marker of severity of acute pancreatitis. The persistent organ failure in acute pancreatitis mainly includes respiratory, cardiovascular, and renal failure [3]. Albumin may improve oxygenation in patients with acute respiratory distress syndrome by reduced alveolar-capillary permeability, decreased inflammatory cell infiltration, enhanced total plasma antioxidant capacity, and faster hemodynamic stabilization [35, 36]. A study in chronic obstructive pulmonary disease suggested that hypoalbuminemia is independently associated with the development of acute respiratory failure [37]. On the other hand, hypoalbuminemia is associated with decreased colloid osmotic pressure, which can lead to the development of pulmonary edema and exacerbation of acute heart failure [38]. The causal relationship between hypoalbuminemia and acute kidney injury is now confirmed [39]. Albumin may improve renal perfusion and glomerular filtration through prolonged renal vasodilation [40, 41], stimulation of the proliferation of proximal renal tubular epithelial cells by activation of mitogenic pathways [42], and inhibition of apoptosis of renal tubular cells by scavenging of reactive oxygen species [43]. Therefore, the fact that hypoalbuminemia is associated with prolonged hospital stay and increased morbidity and mortality has been noted in many clinical scenarios regardless of the implicated disease [10, 11, 13, 15, 16, 27]. On the other hand, positive protective effects of human albumin solutions have been noted in several clinical scenarios such as septic shock [44] and liver cirrhosis with hepatorenal syndrome [45] or with spontaneous bacterial peritonitis, a disease that shares important pathophysiological features with severe sepsis [46]. It is unknown whether administration of albumin would improve the clinical outcomes of acute pancreatitis with hypoalbuminemia though severe acute pancreatitis has many similarities to sepsis syndrome and septic shock [47]. Therefore, it would be interesting to conduct a randomized clinical trial to assess the effect of the correction of hypoalbuminemia on acute pancreatitis in the future.

A few reports have also attempted to study the effect of serum albumin levels on the clinical outcomes of acute pancreatitis. Xue et al. [18] suggested that hypoalbuminemia in the early stage can accelerate the deterioration in pathophysiology of severe acute pancreatitis and it was associated with a high incidence of infection and mortality. Gonzalvez-Gasch et al. [19] use albumin < 25 g/L as a point in the prediction score for complicated acute pancreatitis. A recent study by Li et al. [20], which used persistent organ failure as primary outcome, proposed that serum albumin was a good indicator of persistent organ failure in acute pancreatitis. However, all the above-mentioned have small sample sizes with low statistical power and the results were not adjusted for confounding factors such as age, gender, the time from pain onset to admission, etiology, and chronic concomitant disorders. In addition, no subgroup analysis was performed in these studies. Therefore, these studies were not comparable to our current study. The current study showed that the risk of persistent organ failure significantly increases as levels of serum albumin decrease (*P*_trend_ < 0.001) (Figure 1). Decreased albumin levels were also proportionally associated with prolonged hospital stay (*P*_trend_ < 0.001) and an increased risk of mortality (*P*_trend_ < 0.001). Multivariate logistic regression analysis suggested that an increase of 1 g/L in serum albumin level was associated with a statistically significant 19% (OR: 0.81; 95% CI: 0.76–0.87) reduction in the odds of persistent organ failure after adjusting for potential confounders (Figure 2). Similarly, multivariate logistic regression analysis suggested that an increase of 1 g/L in serum albumin level was associated with a statistically significant 21% (OR: 0.79; 95% CI: 0.69–0.91; *P* = 0.001) reduction in the odds of mortality after adjusting for potential confounders (Figure 3). Subgroup analysis confirmed the strong association between the serum albumin level and persistent organ failure across subgroups of age, gender, the time from pain onset to admission, different etiology, chronic concomitant diseases, total bilirubin, ALT, hematocrit, glucose, and BUN (Figure 4). As to association between the serum albumin level and mortality, subgroup analysis confirmed that these associations were stronger in subgroups of patients with one day from pain onset to admission, nonbiliary etiology, and chronic concomitant diseases than in those subgroups with two or three days from pain onset to admission and biliary etiology and without chronic concomitant diseases (Figure 5).

The area under ROC curves of albumin for predicting organ failure and mortality were 0.78 (95% CI: 0.72–0.85) and 0.87 (95% CI: 0.78–0.95), respectively (Figure 6). These results suggest that albumin may be used as an early predictor of persistent organ failure and mortality. As shown in Table 2, with a cut-off value of ≤30.8 g/L, albumin had a sensitivity of 53% and a specificity of 90% with an overall accuracy of 87% for prediction of organ failure. With a cut-off value of ≤29 g/L, albumin had a sensitivity of 55% and a specificity of 91% with an overall accuracy of 93% for prediction of death. Both cut-off values had low sensitivity, which means that some of these patients who were at high risk of developing persistent organ failure and death may not have a low serum albumin level. However, both cut-off values had high negative predictive value, which is important in emergency for identification of patients not at risk for the development of persistent organ failure and death.

The existing scoring systems have shown moderate accuracy in predicting persistent organ failure but are cumbersome to use in the clinical setting [2]. Xu et al. [48] suggested that fatty liver assessed by computed tomography was associated with severity of acute pancreatitis. Mikolasevic et al. [49] showed that presence of nonalcoholic fatty liver was related to more severe forms of acute pancreatitis. Therefore, it is no surprise that chronic concomitant disease (OR: 5.26; 95% CI: 2.07–11.31) was independently associated with persistent organ failure since fatty liver was the most common chronic concomitant disease in our study (Table 1). Cho et al. [50] reported that patients with alcoholic acute pancreatitis have a longer hospital stay and higher incidence of persistent organ failure compared to those with biliary acute pancreatitis. As expected, our results indicated that patients with biliary etiology had a statistically significant 56% (OR: 0.44; 95% CI: 0.22–0.87) reduction in the odds of persistent organ failure compared to patients without biliary etiology. Simple, routine biomarkers such as hematocrit [7] and blood urea nitrogen (BUN) [7, 51–53] have been proposed as markers of disease severity. Koutroumpakis et al. [7] suggested that admission hematocrit ≥ 44% and rise in BUN at 24 hrs may be the optimal predictive tools in clinical practice among existing laboratory parameters and scoring systems. Mounzer et al. [2] showed that BUN had similar accuracy in comparison with more complex scoring systems in predicting organ failure. As expected, our results suggested that hematocrit ≥ 44% (OR: 2.92; 95% CI: 1.55–5.51) and BUN > 25 mg/dL (OR: 8.30; 95% CI: 4.05–17.01) were independently associated with persistent organ failure. BUN > 25 mg/dL was also associated with a higher mortality risk in acute pancreatitis compared to patients with BUN ≤ 25 mg/dL (OR: 5.06; 95% CI: 1.37–18.67; *P* = 0.015), which is consistent with a previous report [5]. Additionally, contrary to the report by Kumaravel et al. [54], our study showed that BMI was not associated with organ failure and mortality in acute pancreatitis. These differences may be due to the variations among studies regarding the proportion of different etiology and number of obese patients, as evidenced by the fact that only a small proportion of patients with alcohol etiology and obesity were enrolled in our study (Table 1). The drawbacks in currently available data are that risk factors such as blood urea nitrogen are not necessarily pathophysiologically linked to the development of the organ failure and, therefore, elevation of these markers simply reflects ongoing organ failure [9]. Theoretically, pathophysiologically relevant biomarkers may perform better in this clinical setting. In this regard, serum albumin may provide important prognostic information and help risk stratification. The advantage of using of serum albumin levels for predicting outcomes is that it is an objective variable which is easily performed, being inexpensive and available in most hospital laboratories [12].

The strengths of our study include the following: (i) this is the first study to investigate albumin not only as a risk factor but also as a predictor of persistent organ failure and mortality of acute pancreatitis by multivariable logistic regression analysis and subgroup analysis; (ii) exhaustive efforts were made to collect all clinical and laboratory data (including serum albumin and its related confounding factors) in the early stage of acute pancreatitis and a large sample with adequate power enabled us to evaluate the relationship between serum albumin and organ failure of acute pancreatitis; (iii) we have taken serum albumin levels within 24 hours of admission and excluded patients with albumin infusion before data collection so that artifactual influence on serum albumin levels due to intravenous fluid treatment would be minimized [55]. In addition, we also excluded patients with hypoalbuminemia due to malnutrition, chronic renal disease, albuminuria, bleeding/coagulation disorders, chronic alcoholism, hepatitis, and liver cirrhosis. Limitations of our study are as follows. (i) This is a retrospective study, which may have selection bias. Because of the retrospective design, we did not investigate other risk factors of severe acute pancreatitis such as lipase [56], prealbumin/fibrinogen [57], and red cell distribution width [58]. (ii) Data were collected in a single tertiary care hospital, which may not be generalized to a local clinic or a community hospital. Therefore, a prospective external validation of our results is mandatory before its application in the future. (iii) Serum albumin level may have been affected by fluid administration since it was measured within 24 hours of admission [12]. Therefore, another important limitation of current study is that we had no data on the volume of resuscitation or IV fluid therapy due to the retrospective study design. As a remediation, we have evaluated hematocrit and blood urea nitrogen, which often were used as the markers of hemoconcentration [59], as confounding factors of hypoalbuminemia in our multivariate logistic regression analysis.

In summary, as a risk factor, hypoalbuminemia within 24 hrs of hospital admission is independently associated with increased risk of development of persistent organ failure and death in acute pancreatitis. An increase in serum albumin level was associated with a statistically significant reduction in the odds of persistent organ failure and mortality of acute pancreatitis after adjusting for potential confounders. The association between the serum albumin level and mortality was stronger in subgroups of patients with one day from pain onset to admission, nonbiliary etiology, and chronic concomitant diseases than in those subgroups with two or three days from pain onset to admission and biliary etiology and without chronic concomitant diseases. As a predictor, low albumin had a high negative predictive value and allowed identification of patients not at risk of developing persistent organ failure, thereby eliminating the need for intensive care.

## Figures and Tables

**Table 1:** Baseline clinical characteristics and outcomes among patients with different albumin levels.

Characteristic	Normal (*N* = 424)	Hypoalbuminemia		*P* value
		Mild (*N* = 198)	Severe (*N* = 78)	
Median age, years (IQR)	46.5 (37–60)	50 (39–67)	47.5 (36–70)	0.012
Male sex, *N* (%)	273 (64.4)	116 (58.6)	46 (59.0)	0.316
Time from pain onset to admission (days)	1.75 ± 0.77	1.88 ± 0.79	2.10 ± 0.82	<0.001
BMI ≥ 30, *N* (%)	23 (5.4)	12 (6.1)	4 (5.1)	0.934
Etiology^★^				
Biliary, *N* (%)	198 (46.7)	87 (43.7)	23 (30.0)	0.024
Alcohol, *N* (%)	56 (13.2)	29 (14.6)	11 (14.3)	0.888
Hypertriglyceridemia, *N* (%)	21 (5.0)	8 (4.0)	8 (10.1)	0.094
Idiopathic, *N* (%)	145 (34.2)	68 (34.2)	33 (42.9)	0.323
Other, *N* (%)	9 (2.1)	9 (4.5)	4 (5.2)	0.153
Chronic concomitant diseases^*◆*^	240 (56.6)	116 (58.3)	53 (68.8)	0.134
Neurologic, *N* (%)	4 (0.9)	1 (0.5)	5 (0.5)	0.004
Cardiovascular, *N* (%)	13 (3.1)	13 (6.5)	4 (5.2)	0.126
Pulmonary, *N* (%)	5 (1.2)	4 (2.0)	2 (2.6)	0.385
Diabetes mellitus, *N* (%)	66 (15.6)	27 (13.6)	12 (15.6)	0.800
Hypertension, *N* (%)	92 (21.7)	48 (24.1)	20 (26.0)	0.365
Hepatitis virus carrier, *N* (%)	16 (3.8)	7 (3.5)	2 (2.6)	0.721
Fatty liver, *N* (%)	162 (38.2)	63 (31.7)	27 (35.1)	0.279
Laboratory findings				
Hematocrit	0.43 (0.38–0.46)	0.41 (0.37–0.45)	0.40 (0.37–0.45)	0.001
Bilirubin, mg/dL (IQR)	1.17 (0.82–1.70)	1.23 (0.82–1.81)	1.053 (0.64–1.81)	0.805
ALT, U/L (IQR)	43 (21–160)	34 (16–92)	36 (19–57)	0.001
Glucose, mg/dL (IQR)	139 (114–182)	150 (119–200)	171 (117–216)	0.003
BUN, mg/dL (IQR)	12.7 (10.1–16.2)	15.1 (11.2–19.6)	16.0 (10.6–29.4)	<0.001
*Outcomes*				
Median hospital days (IQR)	9 (7–12)	11 (8–14)	13 (9–21)	<0.001
Organ failure *N* (%)	15 (3.5)	20 (10.1)	33 (42.3)	<0.001
Death, *N* (%)	1 (0.2)	3 (1.5)	7 (9.0)	<0.001

^★^Subjects may have more than one etiology. ^*◆*^Subjects may have more than one chronic concomitant disease.

**Table 2:** The best cut-off values of albumin levels to predict organ failure and death in patients with acute pancreatitis.

	Organ failure	Death
AUC (95% CI)	0.78 (95% CI: 0.72–0.85)	0.87 (95% CI: 0.78–0.95)
Albumin level (cutoff point)	≤30.8 g/L	≤29 g/L
Sensitivity	0.53	0.55
Specificity	0.90	0.92
Positive likelihood ratio	5.40	6.83
Negative likelihood ratio	0.52	0.49
Positive predictive value	0.37	0.10
Negative predictive value	0.95	0.99
Diagnostic accuracy	0.87	0.91

AUC = area under the curve of the Receiver Operating Characteristic Curve.
